# Autophagic degradation of CNS myelin maintains axon integrity

**DOI:** 10.15698/cst2022.12.274

**Published:** 2022-11-21

**Authors:** Niki Ktena, Stefanos Ioannis Kaplanis, Irina Kolotuev, Alexandros Georgilis, Emmanouela Kallergi, Vasiliki Stavroulaki, Vassiliki Nikoletopoulou, Maria Savvaki, Domna Karagogeos

**Affiliations:** 1 School of Medicine, University of Crete, Heraklion, Greece.; 2 Institute of Molecular Biology and Biotechnology, FORTH, Heraklion, Greece.; 3 Electron Microscopy Facility (PME), University of Lausanne, Lausanne, Switzerland.; 4 Department of Fundamental Neurosciences (DNF), University of Lausanne, Lausanne, Switzerland.

**Keywords:** autophagy, myelin, CNS, oligodendrocyte, PLP, MBP

## Abstract

(Macro)autophagy is a major lysosome-dependent degradation mechanism which engulfs, removes and recycles unwanted cytoplasmic material, including damaged organelles and toxic protein aggregates. Although a few studies implicate autophagy in CNS demyelinating pathologies, its role, particularly in mature oligodendrocytes and CNS myelin, remains poorly studied. Here, using both pharmacological and genetic inhibition of the autophagic machinery, we provide evidence that autophagy is an essential mechanism for oligodendrocyte maturation *in vitro*. Our study reveals that two core myelin proteins, namely proteolipid protein (PLP) and myelin basic protein (MBP) are incorporated into autophagosomes in oligodendrocytes, resulting in their degradation. Furthermore, we ablated *atg5*, a core gene of the autophagic machinery, specifically in myelinating glial cells *in vivo* by tamoxifen administration (*plp-Cre*^*ERT2*^*; atg5* ^*f/f*^) and showed that myelin maintenance is perturbed, leading to PLP accumulation. Significant morphological defects in myelin membrane such as decompaction accompanied with increased axonal degeneration are observed. As a result, the mice exhibit behavioral deficits. In summary, our data highlight that the maintenance of adult myelin homeostasis in the CNS requires the involvement of a fully functional autophagic machinery.

## INTRODUCTION

Myelin is the multilamellar, lipid-rich membrane that wraps the majority of vertebrate axons and ensures the rapid action potential propagation over long distances. Furthermore, this evolutionary innovation of vertebrates, serves as an insulator of the axon and participates in axon trophic support [1, 2]. In the central nervous system (CNS), myelin is produced by the membrane extension of specialized glial cells, the oligodendrocytes (OL). In CNS myelin, the most abundant proteins are the transmembrane proteolipid protein (PLP, 50% of CNS myelin proteins) and the small cytoplasmic myelin basic protein (MBP, 30%) [3]. Both are of utmost importance for myelin compaction with the former mediating compaction on the extracellular side of the membrane and the latter involved in myelin compaction between two cytoplasmic membrane leaflets. Genetic defects in myelin genes as well as disruption of myelin membranes in adulthood lead to severe motor and/or cognitive impairments in humans. Furthermore, myelin damage is linked to neurodegenerative diseases including multiple sclerosis, Alzheimer's, Parkinson's disease and others [4, 5].

Recent work has contributed to the notion that myelin is not a static structure, as initially considered, but it undergoes constant turnover throughout life to retain its plasticity [6, 7]. It has been shown that there is a constant replenishment and degradation of myelin constituents in order to avoid functional decline of the membrane [8, 9, 10]. However, the mechanism via which myelin and myelin proteins are degraded during this remodeling remains unknown. A very recent study pointed to the role of microglia that phagocytose myelin sheaths during developmental myelination [11], although microvascular endothelial cells and astrocytes have also been previously reported to engulf myelin debris [12, 13].

Macroautophagy (hereafter referred to as autophagy) comprises an evolutionarily conserved pathway delivering proteins and damaged organelles to lysosomes for degradation [14]. It initiates with the engulfment of intracellular cargo by the phagophore, which eventually expands to form a double-membrane vesicle, termed the autophagosome. The whole process is orchestrated by a network of autophagy related genes (ATGs), such as the ATG12 conjugation system (Atg12-Atg5-Atg16), that promotes the formation of MAP1LC3/LC3 (microtubule associated protein 1 light chain 3)-positive phagophores [15, 16]. Following this step, the autophagosome will finally fuse with the lysosome to form a structure called autolysosome, which constitutes the final step of autophagy and is responsible for the degradation [16, 17].

Though initially perceived as a non-selective process, emerging evidence clearly suggests that it can be highly selective and serve specific functions by degrading diverse substrates, including lipid droplets (lipophagy), aggregated proteins (aggrephagy), or mitochondria (mitophagy) [18]. Recently, a new type of selective autophagy in Schwann cells was described, which mediates the degradation of myelin surrounding peripheral nerves after injury (myelinophagy; [19]). The authors showed that activation of autophagy is an early and essential step for myelin clearance after trauma and subsequent axonal regeneration. Similar studies support the Schwann cell autophagy-mediated myelin clearance [20, 21, 22]. In contrast to peripheral nervous system (PNS) and Schwann cells, the role of autophagy in CNS and OLs is not completely understood. Recent work has demonstrated that autophagy in OLs is involved in both CNS injury and recovery [23, 24]. Moreover, it has been recently suggested that autophagy plays a key role in the survival and differentiation of OL progenitor cells and proper myelin development [25].

The aim of this study was to elucidate the role of autophagy in mature OLs and in CNS myelin homeostasis. Our findings strongly support that autophagy is an essential mechanism for OL maturation and maintenance during adulthood. More specifically, we demonstrate for the first time that autophagy plays a crucial role in maintaining cellular homeostasis in adult CNS as a basic degradative mechanism for myelin proteins and this mechanism is supported by our *in vitro* data as well. Upon ablation of autophagy *in vivo*, myelin proteins accumulate, which leads to severe morphological and behavioral defects in mice. Taken together, these results provide novel insights into alternative mechanisms of CNS myelin degradation, other than the canonical processes mediated by microglia and astrocytes.

## RESULTS

### Inhibition of autophagy results in maturation defects in oligodendrocytes

**Figure 1:**
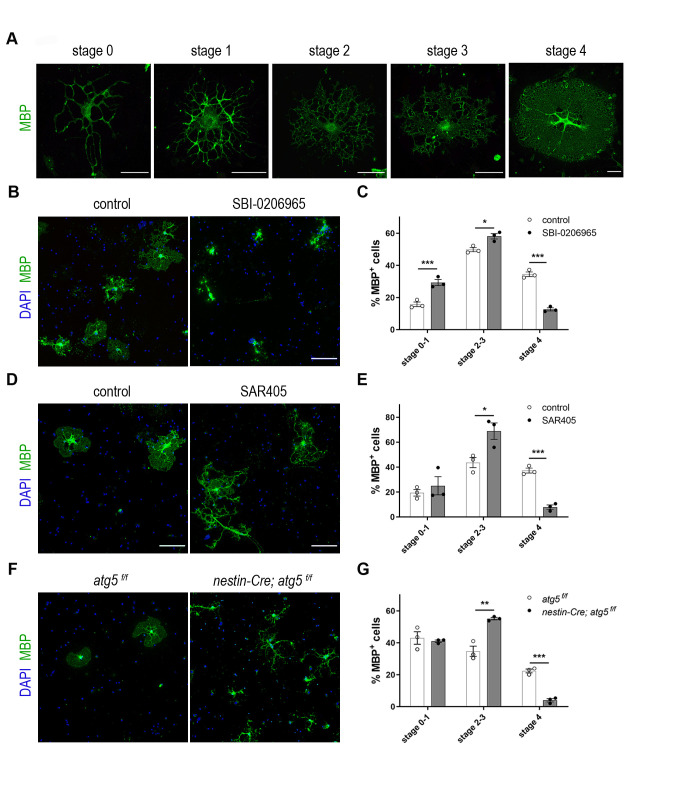
FIGURE 1: Pharmacological and genetic inhibition of autophagy results in maturation defects in oligodendrocytes. (A) Representative examples of mature DIV5 MBP-expressing OLs (MBP in green) that can be subdivided in five maturation stages. Scale bars: 30 μm. (B) Representative confocal images of DIV5 primary oligodendrocytes (OLs), immunolabeled for MBP (green) and DAPI (blue). Cells were either vehicle treated (control), or treated for 5 days with 1μM SBI-0206965. Scale bars: 100 μm. (C) Quantification of the percentage of the MBP+ OLs found in the stages described in the text. (D) Representative confocal images of cultured DIV5 primary oligodendrocytes (OLs), immunolabeled for MBP (green) and DAPI (blue). Cells were either vehicle treated (control), or treated for 5 days with 1μM SAR405. Scale bars: 100 μm. (E) Quantification of the percentage of the MBP+ OLs found in the categories described in the text. (F) Representative confocal images of DIV2 primary control (*atg5* ^*f/f*^) and cKO (*nestin-Cre; atg5*^*f/f*^) OLs immunolabeled for MBP (green) and DAPI (blue). Scale bars: 100 μm. (G) Quantification of the percentage of the MBP+ OLs found in the categories described in the text. Data information: Data are shown as mean ± SEM. N=3 independent experiments per condition. An average of 170-200 cells per treatment per experiment were analyzed. For the quantification of OL complexity, Student's t-test was used to determine statistical significance between control and treated/cKO cells of each stage. *p < 0.05, **p ≤ 0.01, ***p ≤ 0.001.

In an effort to understand the importance of the autophagy pathway in CNS myelin, we first tested its effects in the maturation of OLs. For this reason, we established primary OL cultures derived from newborn P2 C57BL/6 mice, where MBP+ OLs were analyzed. MBP+ mature OLs can be categorized into five morphological stages (stages 0-4), according to their maturation index. Specifically, according to the literature [26], we identified: stage 0-1 as cells with primary or multiple processes and without myelin membranes, stage 2-3 cells with increased ramification branching and the initiation or extension of myelin sheet formation and finally, stage 4 OLs, which represent cells at their final maturation that display a fully developed myelin membrane sheet surrounding all the cell branches **(Figure 1A)**. In culture, with increasing days *in vitro* (DIV), higher percentages of differentiated oligodendrocytes are detected with a fully developed myelin sheet (stage 4 OLs).

We initially performed pharmacological blockage of autophagy, using a potent and selective inhibitor of the serine/threonine autophagy-initiating kinases ULK1, named SBI-0206965 [27] **(Figure 1B)**. After DIV5, these cultures were immunostained for MBP and DAPI and observed with confocal microscopy. Upon pharmacological blockage of autophagosomal formation, we noticed maturation defects in SBI-treated cells since there were fewer OLs reaching final maturation stage 4 **(Figure 1C)**. In parallel, we detected an increase in the proportion of early stage OLs (stage 0-1 and 2-3 OLs) in SBI-treated cultures. These results were further validated by a ULK1-independent inhibitor, namely SAR405. SAR405 is a PIK3C3/Vps34 inhibitor that selectively prevents autophagy [28]. The evaluation of the effects of this inhibitor upon OL maturation was performed similarly to SBI-0206965 and showed similar results **(Figure 1D, E)**. Caspase-3 immunostaining of cultures treated with either SBI-0206965 or SAR405 showed no toxic effects on cell viability in both cases **(Figure S1A-D)**. Moreover, protein levels of S318-ATG13 (normalized over the total ATG13 levels), a downstream target of ULK1 kinase, were found decreased in the case of SBI-0206965 treatment, as expected **(Figure S1E)**. Finally, normalized protein levels of LC3-II form were decreased in cultures treated either with SBI-0206965 or with SAR405, as expected **(Figure S1F, G)**.

In order to corroborate the importance of autophagy in OL maturation, we decided to examine primary OLs from mice in which autophagy is genetically ablated. To this end, we prepared primary OL cultures from P2 *nestinCre; atg5* ^*f/f*^ mice [29] and littermate controls **(Figure 1F)**. The nestin promoter drives Cre recombinase expression in neuronal and glial cell precursors. Similar to the pharmacological blockage, genetic ablation of *atg5* in pure oligodendrocytic cultures demonstrated maturation defects in mutant cells **(Figure 1G)**.

### Autophagy depletion leads to PLP accumulation *in vivo*

**Figure 2:**
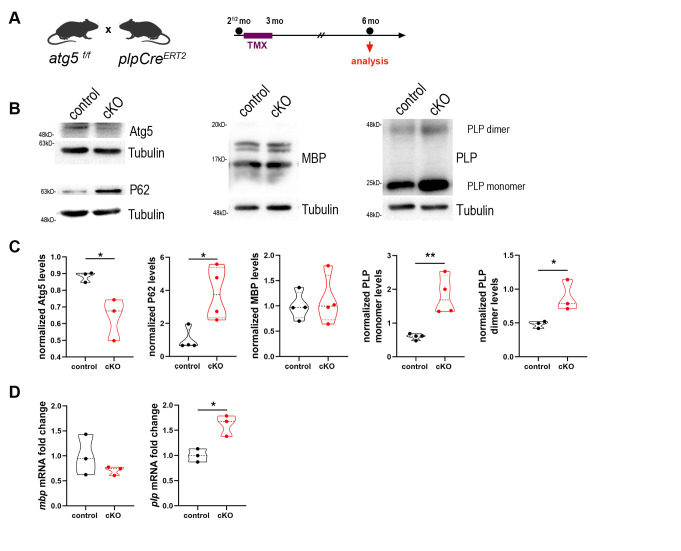
FIGURE 2: Blockage of autophagy leads to increased plp mRNA and protein levels. (A) Schematic illustration of the experimental protocol used for tamoxifen (TMX) induction and analysis in the *plpCre*^*ERT2*^*; atg5* ^*f/f*^ mice. All animals were injected i.p. with 1 mg of TMX per day at the age of 2.5 months for 10 days with two days break in between. The analysis was performed at 6 mo. (B) Western blot analysis with antibodies against Atg5, P62, PLP and MBP proteins in optic nerve lysates of 6mo control and cKO mice. Representative images of a single experiment are depicted. (C) Quantification of normalized protein levels of “B”. (D) Quantification of quantitative real-time PCR in mouse optic nerves of 6mo control and cKO mice. Data information: Data are shown as mean ± SEM. N=3-4 animals per genotype. Student's t-test was used to determine statistical significance between control and cKO protein and mRNA levels. *p < 0.05, **p ≤ 0.01.

We further investigated the role of autophagy in OLs in an *in vivo* model. To this end, and in order to study the contribution of autophagy in mature OLs specifically, we crossbred the *atg5* ^*f/f*^ mice with tamoxifen-inducible *plpCre*^*ERT2*^ mice in order to eliminate the core autophagic component *atg5* exclusively in myelinating glial cells after tamoxifen administration. We first verified the successful recombination and the pattern of Cre activity by crossing our line with a transgenic reporter mouse line, namely mT/mG [30]. In this line, tdTomato (*mT*) fluorescence expression is widespread in cells, while the red fluorescence is replaced by the cell membrane-localized enhanced green fluorescent protein (EGFP) (*mG*), in Cre recombinase-expressing cells. Following the same experimental design used in our autophagy depleted mice *(plpCre*^*ERT2*^; *atg5* ^*f/f*^) **(Figure S2A)**, and as shown in confocal images of both optic nerve sections **(Figure S2B)** and sagittal brain sections **(Figure S2C)** of tamoxifen-injected *mT/mG*; *plpCre*^*ERT2*^- and *mT/mG*; *plpCre*^*ERT2*^+ mice, EGFP expression is present strictly in myelin tracts of the latter, and absent in *mT/mG*; *plpCre*^*ERT2*^- tamoxifen-injected littermates. After verifying the efficiency of the recombination, we injected our mice (*plpCre*^*ERT2*^; *atg5* ^*f/f*^) following the same tamoxifen administration protocol at 2.5mo and performed analysis on 6mo control (*atg5* ^*f/f*^) and cKO (*plpCre*^*ERT2*^*+*; *atg5* ^*f/f*^*)* animals **(Figure 2A)**. Mice of both genotypes were treated with tamoxifen. The sufficiency of autophagic depletion in cKO mice was further validated via western blot analysis by both decreased Atg5 levels and at the same time increased levels of the autophagic receptor p62, compared to controls **(Figure 2A, C)**.

First and foremost, we aimed to evaluate whether the main protein constituents of myelin, namely PLP and MBP, are altered in the mutants. Noticeably, we found that PLP protein levels were increased more than 2.5-fold as shown in the western blot analysis of the 6mo cKO mice, while MBP levels did not show any difference **(Figure 2B, C)**. These data indicate that the autophagic machinery could be essential for the degradation of the PLP protein since its dysregulation leads to accumulation of PLP. Since increased levels of PLP protein could also arise from a transcriptional dysregulation of the gene, we performed quantitative real-time PCR on optic nerve extracts from 6mo control and cKO animals and measured the mRNA levels of *plp* and *mbp* genes, which showed a similar trend as the protein levels. In other words, *plp* mRNA was increased 1.5-fold in cKO compared to controls, while there was no difference in *mbp* mRNA levels between the two groups **(Figure 2D)**.

In the literature, increased copies in PLP1 gene and subsequently in PLP protein lead to an X-linked inherited demyelinating disorder, that of Pelizaeus-Merzbacher disease (PMD). PMD is a dysmyelinating and neurodegenerative genetic disorder which affects patients CNS. The disease, resulting from mutations in the PLP1 gene, shows a great degree of heterogeneity in humans with duplication of the gene being a frequent cause leading to severe neurological symptoms [31]. Furthermore, it is known that mutant forms of PLP identified in patients of PMD, can form homo-oligomers and this formation may contribute to the pathophysiology of the disease [32]. To test this hypothesis, we also examined the dimeric form of PLP protein, which also is increased in the cKOs, compared to controls **(Figure 2B, C)**.

We subsequently asked whether PLP accumulation could be toxic to OL populations, as it occurs in PMD models [33, 34]. In order to study the influence of autophagy ablation in different OL populations, we performed immunohistochemistry analysis against CC1 (mature OLs) and PDGFRa (oligodendrocyte progenitor cells, OPCs) in coronal sections of rostral corpus callosum of 6mo control and cKO mice **(Figure S3A)**. In this myelin-mature system, no differences were detected in the numbers of the different subpopulations between the two groups **(Figure S3B)**. The dispensable role of Atg5 in cell survival was further confirmed by the absence of cleaved caspase-3 activity in the region of the corpus callosum, a region rich in somata of OLs **(Figure S3C)**. These results indicate that loss of autophagy in mature OLs does not cause their death for at least 3 months following Atg5 deletion.

### Myelin proteins are part of autophagic cargo

**Figure 3:**
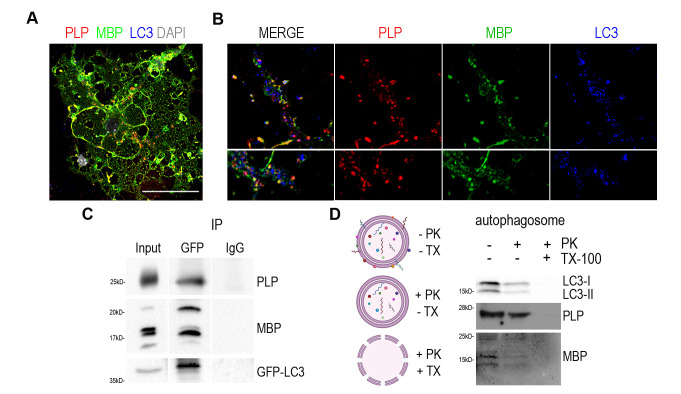
FIGURE 3: Myelin proteins PLP and MBP are detected in the autophagosomes. (A) Representative confocal image of a DIV2 primary OL, immunolabeled for MBP (green), PLP (red), LC3 (blue) and DAPI (grey). Rectangular boxes indicate areas magnified to the right. Scale bar: 30 μm. (B) Magnification images are of a single z-stack (0,5μm thick) and white arrows indicate the colocalized puncta in myelin membrane (top) and cytoplasmic processes (bottom). Scale bars: 2 μm. (C) Immunoprecipitation with antibodies against GFP in forebrain lysates from adult GFP-LC3 mice identifies MBP and PLP as interactors of LC3. (D) Shematic representation of the proteinase K protection assay and western blot analysis of isolated autophagosomes after proteinase K assay. Triton X-100 (TX-100) is used as a negative control. Autophagic marker LC3-II was protected from PK digestion, unless TX-100 was present. Both PLP and MBP seem to be protected from PK treatment.

We set the hypothesis that autophagy may serve to degrade myelin proteins and through the ablation of this degradation in adult CNS, PLP protein accumulates *in vivo.* First, we aimed to validate the direct interaction of myelin proteins with autophagic vesicles. To this end, we performed primary OL cultures and examined the expression of the two most abundant protein components of CNS myelin, namely PLP and MBP, compared to the autophagosomal marker LC3-II **(Figure 3A)**. As shown in higher magnifications, both proteins seem to be engaged in LC3-positive puncta in both the myelin membrane and cytoplasmic processes **(Figure 3B)**. Although MBP protein does not accumulate upon autophagy ablation in contrast to PLP **(Figure 2B, C)**, we continued to further test both myelin proteins interaction with LC3-II protein, *in vivo* this time, and performed immunoprecipitation experiments using forebrain lysates from the GFP-LC3 transgenic line **(Figure 3C)**. We found that both PLP and MBP proteins co-immunoprecipitated with fused LC3. Moreover, in a parallel unbiased approach [35], autophagic vesicles were isolated from adult wild type mouse forebrains and further assessed to a Proteinase K (PK) digestion step in order to distinguish between the autophagosome cargo and the proteins attached to the outer membrane of the vesicle. Protein cargoes tend to be protected from the addition of PK, unless the nonionic detergent Triton X-100 is present **(Figure 3D)**. As shown in Figure 3D both proteins were protected in case of PK treatment and only digested in the presence of the Triton X-100 detergent, suggesting that they are protein cargoes engulfed in autophagosomes.

**Figure 4:**
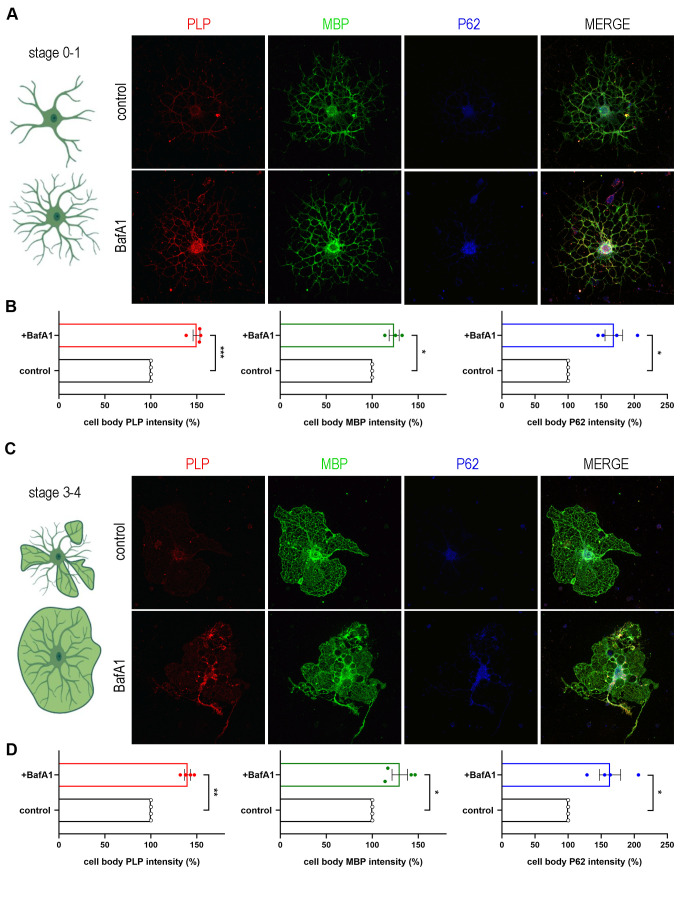
FIGURE 4: PLP and MBP constitute autophagic cargoes. A, C. Confocal images of DIV2 primary OLs, stage 0-1(A) and stage 3-4 (C), immunolabeled for PLP (red), MBP (green) and P62 (blue). Cells were either vehicle treated (control), or treated for 4 h with 10nM BafilomycinA1 (BafA1). B, D. Quantification of the normalized intensity levels of the above-mentioned proteins. Data information: Data are shown as mean ± SEM. N=4 independent experiments. 120-170 cells of stage 0-1 (B) and 40-50 cells of stage 3-4 (D) were used for the analysis. Student's t-test was used to determine statistical significance. *p < 0.05, **p ≤ 0.01, ***p ≤ 0.001. Scale bars: 30 μm.

In order to confirm whether these two structural proteins are indeed cargo of the autophagosome and are targeted to lysosomes, we blocked the final step of the autophagic pathway, therefore preventing the lysis of the constituents of the autophagic vesicles. For this reason, we treated cultured primary OLs with Bafilomycin A1 (BafA1), an inhibitor of lysosome acidification. To improve the reliability of the analysis, we subdivided the OLs in two different groups, namely stage 0-1 (OLs without myelin membranes, **Figure 4A**) and stage 3-4 (OLs with myelin membranes, **Figure 4C**), since there are significant differences in the signal intensity of proteins between those groups. Treatment conditions in this experiment were tested and no cell toxicity of BafA1 was detected **(Figure S4)**. Noticeably, BafA1-treated cells exhibited increased levels of p62 signal, a well-known substrate of autophagy, in both groups of OLs **(Figure 4B, D)**. In parallel, we observed that beyond p62, PLP and MBP also accumulated in the soma of OLs upon BafA1 treatment in both stage 0-1 and stage 3-4 groups, indicating that these proteins use the autophagic machinery for their degradation **(Figure 4B, D)**.

To further confirm these results, we sought to identify the potential existence of LC3 interaction region (LIR) motifs in our myelin proteins, using the ‘‘iLIR'' server for in silico identification of functional LIR motifs [36, 37]. The presence of such short linear motifs allows predicting the interaction between any given protein of interest and Atg8-family members. The results are sorted either as an extended LIR-motif (xLIR), or “canonical” LIR motif (WxxL), where “x” represents any amino acids with the only restrictions for W (W/F/Y) and L (L/I/V) positions. Using this database, LIR motifs (WxxL) were in silico identified in both mouse PLP and MBP **(Table S1)**, further strengthening our *in vitro* and *in vivo* results.

### Autophagy in oligodendrocytes is an essential mechanism for the maintenance of CNS myelin and its depletion causes behavioral deficits

**Figure 5:**
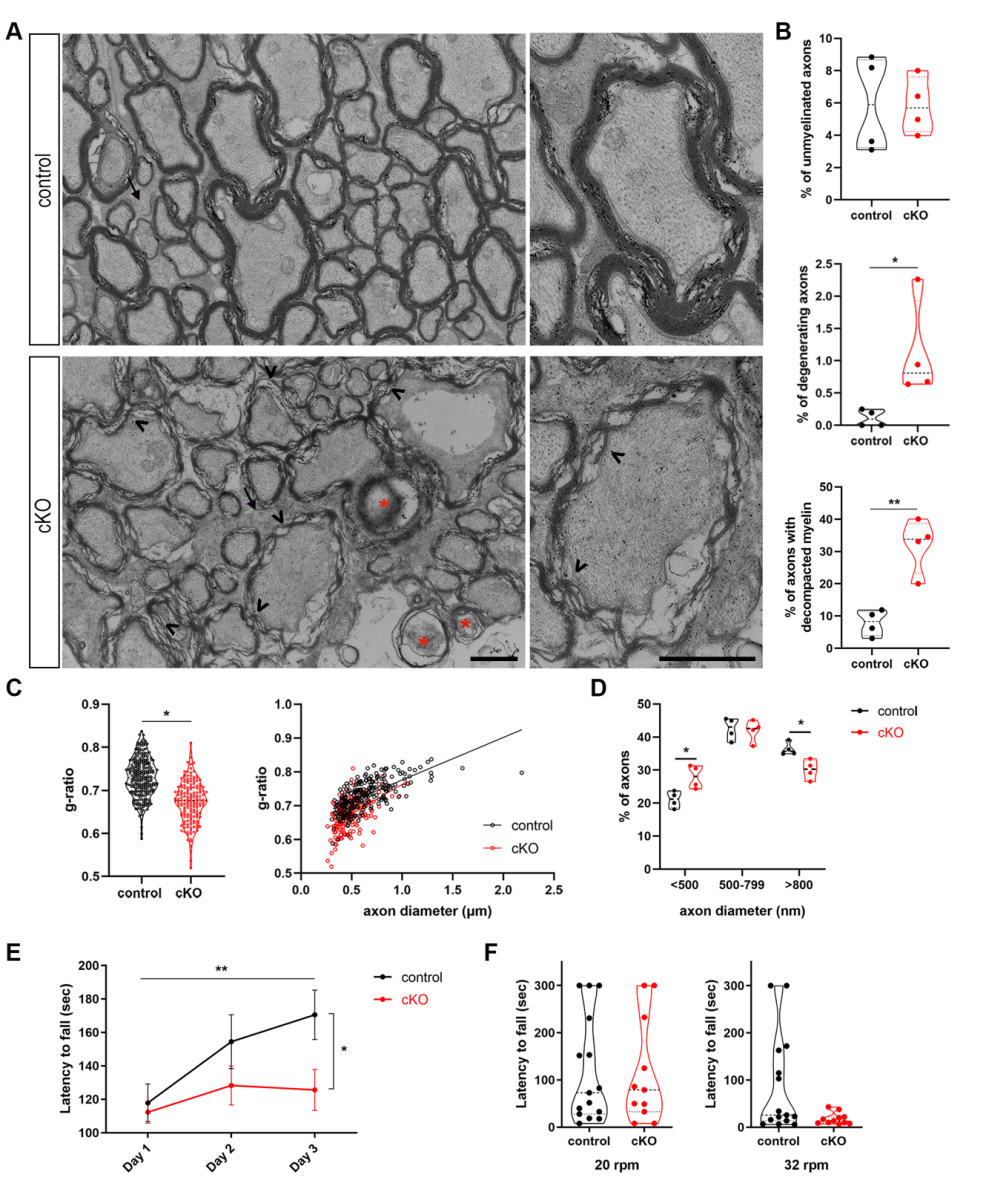
FIGURE 5: Autophagy in OLs is an essential mechanism for the maintenance of CNS myelin. (A) Ultrastructural analysis of optic nerves from *plpCre*^*ERT2*^*-; atg5* ^*f/f*^ (control) and *plpCre*^*ERT2*^*+; atg5*^*f/f*^ (cKO) mice. Representative electron micrographs of cross sections of control and cKO optic nerves, demonstrating the presence of degenerating axons (indicated by red stars), decompacted myelin sheaths (arrowheads) and unmyelinated axons (arrows). (B) Quantification of axonal degeneration, decompacted myelin sheaths and unmyelinated axons in control and cKO mice. Violin plots presenting individual values (450-500 axons measured for each mouse, student's t-test. N= 4 animals per genotype). (C) Myelin sheath thickness was assessed using g-ratio measurement of EM images from control and cKO animals. Plot of g-ratio values per animal group (each point represents an axon) and g-ratio distribution to different axonal diameters of the two groups (linear regression, 260 measured for the control, 170 axons for the cKO group and n= 4 animals per genotype). Scale bar: 1 μm. (D) Quantitation of the percentage of axons with small (<500nm), medium (500-799nm) and large (>800nm) caliber in control and cKO optic nerves. (250 axons measured for each mouse, student's t-test. N= 4 animals per genotype. *p < 0.05, **p ≤ 0.01. (E) Performance of mice in the rotarod task. Line graph showing latency to fall during training (3 days). N= 15 for control; n=11 for cKO. Two-way ANOVA (Tukey's multiple comparisons test) was used to determine statistical significance. For controls: Day 1 vs. Day 2, p=0.954; Day 2 vs. Day 3, p=0.6255; Day 1 vs. Day 3, p= 0.0093. For cKOs: Day 1 vs. Day 2, p=0.7105; Day 2 vs. Day 3, p=0.9907; Day 1 vs. Day 3, p= 0.7875. For day 3 (control vs cKO), p=0.0362. *p < 0.05, **p ≤ 0.01. (F) Violin plots showing latency to fall during the testing phase in the rotarod task. Control, n= 15; cKO, n=11. Student's t-test was used to determine statistical significance. At Day 4(20 rpm), p>0.9999; at Day 5(32 rpm), p= 0.1052. Data information: Bar graphs depict mean ± SEM.

We have shown so far that both PLP and MBP myelin proteins can be engaged as cargoes by autophagosomes and more importantly that PLP protein accumulates in CNS tissues of adult mice where autophagy is ablated for 3 months. Given the correlation of high doses of PLP protein in the CNS with the neurodegenerative disease PMD, where myelin abnormalities are observed together with late-onset axonopathy [38, 39] and the importance of PLP protein balance with motor and learning behaviors [40], the next question that arose was whether autophagy plays a role in myelin maintenance in the mature CNS (in addition to the PLP homeostasis). In order to test this hypothesis, we performed electron microscopic (EM) analysis of 6mo control and cKO optic nerves **(Figure 5)**. This analysis revealed that in mutant mice there were significantly increased numbers of axons with decompacted myelin, as well as increased numbers of degenerating axons, with the number of unmyelinated axons being unaltered between the two groups **(Figure 5A, B)**. The lack of differences in the number of unmyelinated axons was expected since the ablation of autophagy in these mice is induced after myelination is complete. Additionally, the g-ratio analysis revealed that autophagy-deficient axons had smaller g-ratios, meaning extended myelin sheaths compared to controls **(Figure 5C)**. This extension could partly reflect the loosening of myelin lamellae or a thicker myelin sheath *per se*. Finally, analysis of axon diameters showed that there was a small but statistically significant redistribution of the percentages of axon caliber diameters, with the 6mo cKO axons presenting a loss of large caliber axons and increase in the pool of small caliber axons **(Figure 5D)**. The above results, indicate that large caliber axons are more vulnerable in case of autophagic dysfunction and due to PLP accumulation. Larger diameter axons are needed to achieve greater conduction velocity and thus shorter conduction times [41, 42]. Furthermore, it has been proposed that large caliber axons can support larger terminal arbors and more active zones that synaptically transfer information at higher rates [43, 44]. The large caliber axons are also positively associated with thicker myelin sheaths [45], a characteristic that could render them more susceptible to the decompaction and loosening of myelin lamellae, observed in the 6mo cKO mice. Similar myelin abnormalities were revealed in other CNS tracts in our transgenic mice, such as the corpus callosum (data not shown).

Our findings above raise the question whether the blockage of autophagy in OLs, in addition to defects in myelin integrity, also leads to functional impairments. To elucidate this hypothesis, we examined behaviors such as motor learning and motor coordination, which are associated with myelin integrity and axon degeneration, in the brain of 6mo cKO and control mice. To test whether motor learning is altered in case of defective autophagy, mice had to perform an accelerating rotarod test for three consecutive days [46]. In this task, 6mo control mice showed a continuous increase in the time required until they fell within the 3-day trial period, meaning they significantly improved their performance over time. On the other hand, age-matched cKO littermates did not show any statistically significant improvement in their performance over time, indicating that they were not able to learn this skilled motor task **(Figure 5F)**. At day 3, the two groups also revealed statistically significant differences. Following the 3-day accelerating trial **(Figure 5F)**, mice performed the motor coordination testing phase of the task, in which the latency to fall in a rod rotating in a constant speed of 20rpm and 32rpm respectively is tested for the next two days **(Figure 5G)**. A trend of lower latency to fall was detected in cKO mice, only in the higher speed task **(Figure 5G)**, suggesting that 6mo cKO mice have a mild dysfunction in their motor coordination.

Overall, our *in vivo* analysis proposes an important role for autophagy as a homeostatic mechanism for the maintenance of proper PLP levels. Consistently, accumulation of this protein in oligodendrocytes is accompanied by myelin and axon morphological defects as well as defects in cognitive behavior in mice.

## DISCUSSION

Our findings indicate that autophagy is an essential mechanism for OL maturation, since both the pharmacological and genetic inhibition of autophagic vesicle formation led to a final maturation defect in primary OL cultures (fewer stage 4 cells). Furthermore, we showed that the structural myelin proteins PLP and MBP are both engaged and interact with LC3-II in autophagosomes, although only PLP accumulates *in vivo* in case of autophagic blockage. This may be due to the fact that PLP is more abundant in CNS myelin (it consists ∼50% of CNS myelin protein, while MBP about 30% [3]). In addition, MBP may not accumulate because it uses a different mechanism of expression at the site of interest. The majority of MBP protein in translated locally under the OL membrane in contrast to PLP. No excess MBP is detected under the myelin membrane since its translation responds to external stimuli (i.e., axon stimulation) [47]. Thus, cargo MBP for autophagy accounts only for a small portion of the protein, possibly the cytoplasmic and nuclear form and therefore, small differences may not be detected. Alternatively, MBP may be degraded via other mechanisms such as the proteasome that directly takes part in the specific degradation of MBP [48]. Moreover, in the case of PLP, both protein and mRNA levels were significantly increased in case of autophagic depletion in oligodendrocytes. These increased transcriptional levels could imply the possible role of autophagy not only in protein degradation, as is well characterized, but also in mRNA degradation, a pathway that has only very recently been described in yeast [49]. Another potential explanation for this increase could be that the accumulated PLP protein levels are not functional. Hence, the cell may not recognize this nonfunctional PLP, leading to a compensatory increase of *plp* gene transcription. Increased *plp* mRNA expression does not automatically correspond to increased functional PLP protein, as has already been described in the literature in the demyelinating model of PMD [50]. It has been shown that in the PMD mouse model, *plp* gene duplication leads to overexpression of the protein and that the majority of it is degraded through the proteasome [51]. Our study proposes that apart from the proteasome, another major mechanism of degradation for PLP protein is autophagy and that when this mechanism is ablated, PLP accumulates with toxic results for the axons. We suggest that autophagy removes excess or even misfolded PLP protein produced under physiological conditions and during myelin extension in order to retain protein and myelin homeostasis.

Furthermore, in this study we ablated the core autophagic gene *atg5* in OLs after myelination is completed (at 2.5 mo) and analyzed these animals at 6mo. Recent work, targeting OPCs, has shown reduced and defective myelination in case of an early OPC-specific deletion of *atg5* [25]. In our study, in case of late autophagic ablation, we observe significantly increased numbers of axons with decompacted myelin and axonal degeneration. The differences observed in the severity of the phenotype between the two studies may be explained first and foremost due to the fact that Bankston *et al* ablated autophagy in the stage of the progenitors and at an early developmental stage, while in our case, by ablating autophagy in adulthood, we focus on myelin maintenance, not its formation. Secondly, the severity of the phenotype stated in Bankston *et al* may to some degree be attributed to the transgenic line they used, which targets not only OPCs, but also pericytes, cells that are implicated in CNS myelination and whose dysfunction has been recently correlated to white matter changes [52, 53]. Furthermore, our EM analysis revealed a loss of large caliber axons. According to the literature, a shift towards larger axon calibers is observed in ageing optic nerves [54], as well as in pathological cases, such as during chronic secondary degeneration in rat optic nerves [55]. Moreover, 6mo cKO animals present behavioral deficits such as decreased motor skill learning, an ability that is directly correlated to active central myelination [56, 57]. In general, one could notice that this phenotype resembles an “early-ageing” phenotype. In the literature, ageing has been associated with impairments in learning as well as increased degeneration and decompaction of myelin sheaths [58, 59]. In parallel, there is evidence showing that autophagy decreases during ageing, while myelin proteins are increased [60, 61].

Overall, we propose two different hypotheses supported by our results. Firstly, it is possible that all the abnormalities observed in case of ablated autophagy in OLs are a result of PLP accumulation, similar to the case of PMD. In this disease, patients with PLP1 duplication present segmental demyelination along axons, abnormally thick myelin sheaths and degeneration of axons [62], a phenotype that extensively resembles our case. Furthermore, it is noteworthy to mention that in the PMD mouse model, an increased number of autophagosomes has been described which would corroborate our findings [51]. An alternative explanation of this phenotype could be a result of accumulation of damaged or excess myelin membranes *per se*, meaning that autophagy is responsible for degradation of CNS myelin. This hypothesis is supported by the fact that at least two major myelin proteins (PLP and MBP) are shown to interact with autophagosomes, leading to the idea that CNS myelin itself could use this machinery for its degradation. Myelin synthesized during development is continuously exchanged and renewed, through a procedure named myelin turnover, by which new membranes replace old membranes, which then must be removed [7, 63]. Our existing knowledge documents that the elimination of aberrantly deposited myelin -in case of trauma or during development as in myelin turnover- is accomplished through phagocytosis via microglia as well as endocytosis via astrocytes [11, 12, 13]. Thus, our hypothesis is that, in addition to these mechanisms, autophagy in OLs could also clear CNS myelin deposits under steady state conditions, thereby establishing an equilibrium of replenishment and degradation that may also be subject to adaptation with consequences for axonal integrity. This hypothesis does not explain why accumulation of MBP is not detected, unless its different isoforms are undetectable, as mentioned before. In any case, this hypothesis requires further investigation.

## MATERIALS AND METHODS

### Mouse lines

The animal protocols of this study were approved by the Animal Ethics Committee of the Foundation for Research and Technology Hellas (FORTH). All animals were kept at temperature-controlled conditions on a 12 h light/dark cycle, fed by standard chow diet and water ad libitum provided within the animal facility of the Institute of Molecular Biology and Biotechnology (IMBB) - Foundation for Research and Technology Hellas (FORTH) (license nos. EL91-BIObr-01 and EL91-BIOexp-02). All animal experiments complied with the ARRIVE and NC3Rs guidelines to improve laboratory animal welfare and conformed with all regulations and standards outlined in the Presidential Decree 56/30.04.2013 (Greek Law) in accordance with the EU directives and regulations (2010/63/EU and L 276/33/20.10.2010) and to the U.K. Animals (Scientific Procedures) Act, 1986 and associated guidelines, equivalent to NIH standards. The ages of the mice are described in each experiment and only males are used in this study. The animals used were of C57BL/6 genetic background. *Atg5*^*f/f*^ [29], *plpCre*^*ERT2*^ [64] (a generous gift from Dr. Shuter, Zurich), GFP-LC3 (a generous gift from Dr. Chamilos, IMBB-FORTH, initially obtained from RIKEN BioResource Center), *mT/mG* [30] (a generous gift from Dr. Talianidis, IMBB-FORTH) and *nestin-Cre [29]* mouse lines were used. In *plp-Cre*^*ERT2*^*; atg5*^*f/f*^ and *mT/mG; plpCre*^*ERT2*^ progeny, tamoxifen (Sigma-Aldrich, Cat# T5648, dissolved in a sunflower oil:ethanol (9:1) mixture at a concentration of 10mg/ml) was administered by intraperitoneal injections at a dose of 1mg/mouse/day for ten days with a two-day break in between, as previously described [64], starting at 2.5 months (mo) and mice were sacrificed at 6 months (6mo).

### Assessment of mouse motor phenotypes

#### Rotarod test

To assess the acquisition of motor behavior in mice, a rotarod apparatus with automatic timers and falling sensors was used (MK-660D, Muromachi-Kikai, Tokyo, Japan). The training was performed on the apparatus and consisted of four trials per day (with 15 min rests between trials), for 5 consecutive days. In the first three days, mice were placed on the rotating rod at 4 rpm and gradually the speed was increased to 40 rpm. Each trial lasted until the mouse fell from the rod or for a maximum of 300s. In the fourth and fifth day, the task consisted of two consecutive sessions of three trials each (maximum duration, 300s): the first session at a constant speed of 20 rpm, and the second one at 32 rpm. For the analysis the time until the mice dropped from the rod for each of the training days as well as for each testing speed was measured [65, 66].

#### Statistical Analysis

Statistical analyses regarding the behavioral tests were performed using SPSS version 25 (SPSS Inc., Chicago, Illinois). Between-group differences in the tasks in control and cKO mice were examined either with parametric or non-parametric analyses according to normality of the distribution, as examined with the Kolmogorov–Smirnov test. For the rotarod task the effect of genotype on the latency before falling off during training, was assessed using paired t-test. For the 4^th^ (20rpm) and 5^th^ day (32rpm) of the task (testing phase) the effect of genotype on the latency was assessed using one-way ANOVA.

### Primary OL cultures

Primary OL cultures were obtained from postnatal day 2 (P2) mouse cortices (from both male and female pups), as previously described [67]. Briefly, cortices were diced into small pieces, after the removal of meninges, and were mechanically dissociated. Cells were plated onto poly-D-lysine (PDL, Sigma–Aldrich, Cat# A-003-E, 100 mg/ml)-coated 75-cm^2^ culture flasks and cultured in DMEM (Glutamax™, 14,5 g/L d-Glucose, -Pyruvate, ThermoFisher Scientific, Cat# 61965-026), supplemented with 10% FBS (Gibco) and 1% penicillin/streptomycin in an incubator with 5% CO^2^ at 37°C. The culture medium was replenished twice a week, until mixed glial cultures became confluent (after 10–12 days). At this point the microglial cells were removed using an orbital shaker at 200 rpm for 1 h at 37°C and OPC population was separated from the underlying astrocytic cell layer by vigorous shaking (16 hr at 240 rpm, 37°C). OPCs were then washed and seeded at an initial density of 70,000 cells per well in 24-well plates containing 13mm glass coverslips or at an initial density of 35,000 cells per well in 48-well plates containing 9mm glass coverslips. All plates were previously coated overnight with poly-D-lysine (Sigma–Aldrich, Cat# A-003-E). OPCs were cultured in DMEM (Glutamax™, 14,5 g/L d-Glucose, -Pyruvate, ThermoFisher Scientific, Cat# 61965-026), supplemented with 1% N2 (ThermoFisher Scientific, Cat#17502), 1 μM D-biotin (Sigma-Aldrich, Cat# B4501), 1% BSA fatty acid-free (Sigma-Aldrich), 5 μg/ml N-acetylcycteine (Sigma-Aldrich, Cat# A8199), 1% penicillin-streptomycin (ThermoFisher Scientific, Cat# 15070063) and 40 ng/ml T3 (Sigma-Aldrich, Cat# T6397) to allow the immediate differentiation of OPCs toward mature OLs. The day of cell plating is considered as DIV0. Afterward, medium was replaced every other day. For experiments involving SBI-0206965 treatment, SBI-0206965 (Sigma-Aldrich Cat# SML1540, diluted in 100% DMSO, final DMSO concentration in medium 0,1%) was used at a concentration of 1μM (added at DIV0) and replenished every 48 h until DIV5, when cells were fixed or collected for lysis. For experiments involving SAR405 treatment, SAR405 (Sigma-Aldrich Cat# 5.33063, diluted in 100% DMSO, final DMSO concentration in medium 0,1%) was used at a concentration of 1μM (added at DIV0) and replenished every 48 h until DIV5, when cells were fixed or collected for lysis. In case of cell lysates, the medium containing inhibitors/DMSO was replenished 2 h before collecting the cells. Bafilomycin A1 (Sigma-Aldrich Cat# B1793, diluted in 0.1% DMSO) was added in the medium for 4h (10nM) before fixation (at DIV2 OLs). For all treatments, control OLs were treated with the vehicle at the same concentration (0.1% DMSO) and for the same time duration.

### Immunohistochemistry and immunocytochemistry

For immunohistochemistry, brains and optic nerves were harvested after transcardial perfusion with 4% paraformaldehyde (PFA) in 0.1 M phosphate buffer saline (PBS). Tissues were then post-fixed in the same fixative for 30 min at 4°C, cryo-protected overnight in 30% sucrose in 0.1 M PBS, and embedded in 7.5% gelatin/15% sucrose gel. Cryosections of 10 μm-thick for optic nerves and 15 μm -thick sagittal or coronal for brains were obtained and mounted on Superfrost Plus microscope slides (O. Kindler), post-fixed in ice-cold acetone for 10 min, blocked in 5% BSA (Sigma-Aldrich) in 0.1 M PBS for 1h at RT, and incubated with primary (overnight at 4°C) and secondary (2 h at RT) antibodies in 5% BSA, 0.5% Triton-X in 0.1 M PBS.

For immunocytochemistry, cells were fixed with 4% PFA for 15 min at RT, blocked/permeabilized for 30 min with 1% BSA, 0.1% Triton-X in 0.1 M PBS and incubated with the primary antibody and secondary antibodies in 1% BSA, 0.1% Triton-X in 0.1 M PBS. Sections or coverslips were mounted using MOWIOL Reagent (Merk-Millipore, Burlington, MA) and image acquisition was performed in a confocal microscope (TCS SP8, LEICA DMI-8).

The following primary antibodies were used: anti-PLP (1:1000, rabbit, Abcam Cat# ab28486), anti-PLP (1:200, mouse, Invitrogen Cat# MA1-80034), anti-MBP (1:200, rat, Serotec Cat# MCA409S), anti-adenomatous polyposis coli clone CC1 (APC/CC-1) (1:100, mouse, Millipore Cat# OP80) anti-platelet derived growth factor receptor alpha (PDGFRa) (1:100, rat, Millipore Cat# CBL1366), anti-GFP (1:2000, rat, Nacalai Tesque, Cat# 04404-26), anti-p62 (1:500; rabbit, MBL, Cat# PM045), anti-LC3b (1:1000; rabbit, Sigma-Aldrich, Cat# L7543), anti-Caspase 3, active (cleaved) form (1:200; rabbit, Millipore, Cat# AB3623).

Fluorochrome labeled secondary antibodies Alexa Fluor 555 (1:800, anti-mouse, ThermoFisher, Cat# A-21422), CF^®^633 (1:800, anti-rabbit, Biotium, Cat# 20125), CF^®^488A (1:800, anti-rat, Biotium, Cat# 20023, Cy^TM^3 (1:800, anti-rabbit, Jackson ImmunoResearch, Cat# 111-165-003) were also used. DAPI (ThermoFisher, Cat# D1306) was finally used for the visualization of the nuclei.

### Analysis of confocal images

For the measurements of cell fluorescence/intensity (for PLP, MBP, P62, **Figure 4**), immunocytochemistry was performed the same day for all the cells to be analyzed and all images were captured following exactly the same image acquisition settings: ×63 magnification, 0.5 μm step size, and high resolution (1024 × 1024 pixels), identical laser intensity, identical number of z-stacks for each image, using a TCS SP8 laser scanning confocal microscope. Following image acquisition, images were analyzed by Fiji ImageJ software and quantified blindly to the result. In order for the analysis to be more reliable, we subdivided our OLs in two different groups, namely stage 0-1 (OLs without myelin membranes, analyzed 120-170 cells per treatment **Figure 4A**) and stage 3-4 (OLs with myelin membranes, 40-50 cells per treatment analyzed, **Figure 4C**), since there are significant differences in the signal intensity of proteins between those groups. Quantification of fluorescent marker intensity was achieved by measuring the mean fluorescent intensity per soma area using the raw images, as previously described [68]. Background fluorescence was measured for each cell (equals to average fluorescence of surrounding area of each cell) and was subtracted of the corresponding measurement.

### Electron microscopy

#### Tissue preparation

For electron microscopy, mice were perfused with 2.5% glutaraldehyde in 0.1 M phosphate buffer (PB), pH 7.25. Entire dissected optic nerves were placed in the primary fixative overnight at 4°C and then the preparation of the samples for the analysis was done similarly to Pasquettaz *et al [69]*. Briefly, the samples were extensively washed in PB 0.1 M and incubated in 2% (wt/vol) osmium tetroxide and 1.5% (wt/vol) K4[Fe(CN)6] in 100 mM PB buffer for 1h on ice. After an extensive wash with water, samples were incubated for 1h in 1% (wt/vol) tannic acid in 100 mM PB buffer, followed by 1% (wt/vol) uranyl acetate for 2h at ambient temperature. Finally, samples were dehydrated at ambient temperature in gradual ethanol cycles and infiltrated with a mix of ethanol and Epon-Araldite mix (EMS). After several cycles of 100% Epon-Araldite incubations, samples were flat embedded and polymerized for 24h at 60°C [70]. The experimental procedure was performed simultaneously in control and cKO samples, in order to avoid the possible implication of artifacts arising from fixation or embedding.

#### Electron microscopy

Polymerized flat blocks were trimmed using a 90° diamond trim tool (Diatome, Biel, Switzerland). The arrays of 70 nm sections were obtained using a 35° diamond knife (Diatome, Biel, Switzerland) mounted on Leica UC6 microtome (Leica, Vienna). The orientation for the optic nerves was established perpendicular to its length, a cross-section about 1 mm from the optic nerve head. For sectioning, samples were carefully oriented to obtain a perpendicular plane of the optical nerve. In the case of brain slices, the search was targeted to the corpus callosum based on the overall morphology of the processed slice [70, 71]. Sections were collected on polyetherimide-coated carbon slot grids.

TEM samples were analyzed with an FEI CM100 electron microscope (Thermo Fischer Scientific) at 80kV, equipped with a TVIPS camera, piloted by the EMTVIPS program. Images were collected either as single frames or stitched mosaic panels to cover more extensive sample regions.

The multiple tile images were stitched with the IMOD software package (Kremer *et al,* 1996). Data were processed and analyzed using Fiji, IMOD 3dmod, and Photoshop programs.

### Western blot analysis and immunoprecipitation

Tissues were collected and stored at -80°C until their homogenization. The number of animals for each experiment is indicated in the results section. Briefly, tissues were lysed by sonication in RIPA buffer (50mM Tris-HCl pH 8.0, 150mM NaCl, 1% Triton X-100, 0.5% sodium deoxycholate [DOC]) supplemented with protease inhibitor cocktail (Sigma-Aldrich, Cat# P8340) and placed for 20 min on ice, followed by 20 min centrifugation at 18,000 g. In case of cell lysates, primary oligodendrocytes were washed in prewarmed PBS 1X once, collected in RIPA buffer, supplemented with protease inhibitor cocktail for 1 h on ice, sonicated and centrifuged (20 min, 18,000 g, 4°C).

Protein samples were then separated on a 12%, or 15% polyacrylamide gel and transferred to a nitrocellulose membrane (Millipore) for 1 h at 310mA. After blocking for 1 h at room temperature in 5% BSA (Sigma-Aldrich), membranes were incubated in the primary antibodies overnight at 4°C. The following antibodies were used for Western blot (WB) analysis: anti-PLP (1:1000, rabbit, Abcam Cat# ab28486), anti-MBP (1:200, rat, Serotec), Cat# MCA409S, anti-p62 (1:1000; rabbit, MBL, Cat# PM045), anti-LC3_B_ (1:1000; rabbit, Sigma-Aldrich, Cat# L7543), anti-Atg5 (1:1000; rabbit, Novus, Cat# NB110-53818), anti-Atg13 (1:1000; rabbit, Sigma-Aldrich, Cat# SAB4200100), anti-Atg13 pS318 (1:1000; rabbit, Rockland, Cat# 600-401-C49), anti-α-Tubulin, clone DM1A (1:10.000; mouse, Sigma-Aldrich, Cat# T9026).

After three 5 min washes in TPBS (100 mM Na2HPO4, 100mM NaH2PO4, 0.5N NaCl, 0.1% Tween-20), membranes were incubated for 1 h at room temperature in corresponding secondary horseradish peroxidase-conjugated antibodies (Millipore, Cat# AP308P, AP132P, AP189P). Blots were developed by chemiluminescence (Immobilon Classico Western HRP substrate, Merck, Cat# WBLUC0500) according to the manufacturer's instructions. Quantification of band intensity was assessed with the Fiji/ImageJ Gel Analyzer plugin.

For co-immunoprecipitation, SureBeads Protein G Magnetic Beads (Biorad, Cat# 161-4023) were used, following the protocol suggested by the manufacturing company. 1mg of total protein from LC3-GFP adult mouse forebrains with 3 μl of a polyclonal antibody for GFP, was used for each experiment. 3 μl of rabbit IgG control (Abcam, Cat# ab172730) was used in parallel to ensure specificity. Immunopurified material was then used for western blot.

### Biochemical purification of mouse autophagosomes (AVs) and Proteinase K protection assay

Ten cortices and hippocampi of adult (postnatal day 60, P60) C57BL/6J male mice were used to purify brain AVs, as described previously [72]. Briefly, the aforementioned brain areas were homogenized in 10% sucrose, 10 mM Hepes and 1 mM EDTA (pH 7.3) by 20 strokes using a Dounce glass homogenizer. The resulting homogenate was sequentially dilluted with half volume of homogenization buffer (HB) (250 mM sucrose, 10 mM Hepes and 1 mM EDTA, pH 7.3) containing 1.5 mM glycyl-L-phenylalanine 2-napthylamide (GPN). The resulting material was then incubated at 37°C for 7 min, and centrifuged at 2000g for 2 min at 4°C. The nuclear pellet was discarded and the post-nuclear supernatant was loaded on discontinuous Nycodenz gradients for centrifugation at 141.000g for 1h at 4°C, to remove the cytosolic, mitochondrial and peroxisomal fraction. The isolated layer of material, which contained both autophagosomes and endoplasmic reticulum, was further diluted with an equal volume of HB buffer and overlaid on Nycodenz-Percoll gradients. After centrifugation at 72000g for 30 min at 4°C to remove the Percoll silica particles, the resulting interface that contained the AVs was diluted with 0.7 volumes of 60% Buffered Optiprep overlaid by 30% Buffered Optiprep and HB buffer. The gradients were then centrifuged at 71000g for 30 min at 4°C. The collected AVs were diluted in three volumes of HB buffer and the concentration of the purified isolated AVs was then measured by BCA, following the manufacturer's instructions. For the Proteinase K (PK) protection assay, AVs were treated with PK (20ng/μl) on ice for 20 min, in the presence or absence of 1% Triton X-100, and then PK was inactivated using 4 mM of PMSF for 10 min on ice. The AVs were then centrifuged at 16.900g for 10 min at 4°C, and the autophagosomal pellets were resuspended in Laemmli buffer and boiled at 95°C for 5 min for immunoblotting analysis. The purity of autophagosomes isolated out of this protocol and the absence of contamination by other organelles is already established [35].

### Real time PCR

Total RNA was prepared from optic nerve samples from control and cKO animals (3 animals/group) by using RNAiso-plus kit (Takara) and according to the manufacturer's instructions. cDNAs were synthesized through reverse transcription from the total RNA according to the protocol of PrimeScript 1st strand cDNA synthesis kit (Takara, Cat. #6110A). Expression levels of genes encoding plp (forward primer: 5-TCAGTCTATTGCCTTCCCTA-3, reverse primer: 5-AGCATTCCATGGGAGAACAC-3), mbp (Pernet *et al,* 2008; forward primer: 5-CACACACGAGAACTACCCA-3, reverse primer: 5-GGTGTTCGAGGTGTCACAA-3), were examined by real-time PCR analysis using a StepOnePlus real-time PCR system (Applied Biosystems, Life Technologies, Thermo Fisher Scientific Inc., Waltham, MA). Gapdh was used as the internal control (forward primer: 5'-ATTGTCAGCAATGCATCCTG-3', reverse primer: 5'-ATGGAC TGTGGTCATGAGCC-3'). PCR runs were performed for each sample in triplicates and the expression levels for each gene were normalized according to the internal control.

### Statistical analysis

In all experiments, data were expressed as mean ± SEM. Parametric tests including two-tailed, unpaired t test and one-way analysis of variance (ANOVA), followed by Tukey post-hoc test for multiple comparison procedures were performed where values follow a Gaussian (normal) distribution. In the case of non-normally distributed values, non-parametric Mann–Whitney test or Kruskal–Wallis test with Dunn's post hoc tests for multiple comparisons were used. Statistical analysis, in all the experiments except from the behavioral tests, was performed using GraphPad Prism 8 software (GraphPad Software, San Diego, CA). P-values < 0.05 were considered statistically significant.

### Bioinformatic analysis

For the LIR motif analysis of the MBP and PLP myelin proteins the iLIR Autophagy database was used (https://ilir.warwick.ac.uk/).

## SUPPLEMENTAL MATERIAL

Click here for supplemental data file.

All supplemental data for this article are available online at https://www.cell-stress.com/researcharticles/2022a-ktena-cell-stress/.

## Abbreviations:

CNS: – central nervous system,
OL: – oligodendrocytes,
PLP: – proteolipid protein,
MBP: – myelin basic protein,
ATGs: – autophagy related genes,
MAP1LC3/LC3: – microtubule associated protein 1 light chain 3,
mT: – tdTomato,
mG: – membrane-localized EGFP,
PMD: – Pelizaeus-Merzbacher disease,
OPCs: – oligodendrocyte progenitor cells,
PK: – Proteinase K,
BafA1: – Bafilomycin A1,
LIR: – LC3 interaction region,
EM: – electron microscopic,
DIV: – days in vitro,
EGFP: – enhanced green fluorescent protein,
PNS: – peripheral nervous system
