# Supplementary material for: Autophagic degradation of CNS myelin maintains axon integrity
Source: Cell Stress. 2022 Nov 21;6(12):93–107. doi: 10.15698/cst2022.12.274 (PMC9707329; doi:10.15698/cst2022.12.274)
Supplement: Supplementary file 1 [file ces-06-093-s01.pdf]

Supplemental Informaton

Autophagic degradation of CNS myelin maintains axon integrity

Niki Ktena[1], Stefanos Ioannis Kaplanis[1], Irina Kolotuev[2], Alexandros Georgilis[1], Emmanouela Kallergi[3], Vasiliki Stavroulaki[1], Vassiliki Nikoletopoulou[3], Maria Savvaki[1,*] and Domna Karagogeos[1,*]

[1] School of Medicine, University of Crete, Heraklion, Greece.
[2] Electron Microscopy Facility (PME), University of Lausanne, Lausanne, Switzerland.
[3] Department of Fundamental Neurosciences (DNF), University of Lausanne, Lausanne, Switzerland.

* Corresponding Authors:
Maria Savvaki, PO Box 1385, Vassilika Vouton, IMBB, Heraklion, GR-71110, Greece; E-mail: msavaki@imbb.forth.gr
Domna Karagogeos, PO Box 1385, Vassilika Vouton, IMBB, Heraklion, GR-71110, Greece; Phone +30-2810-394542; E-mail: karagoge@imbb.forth.gr

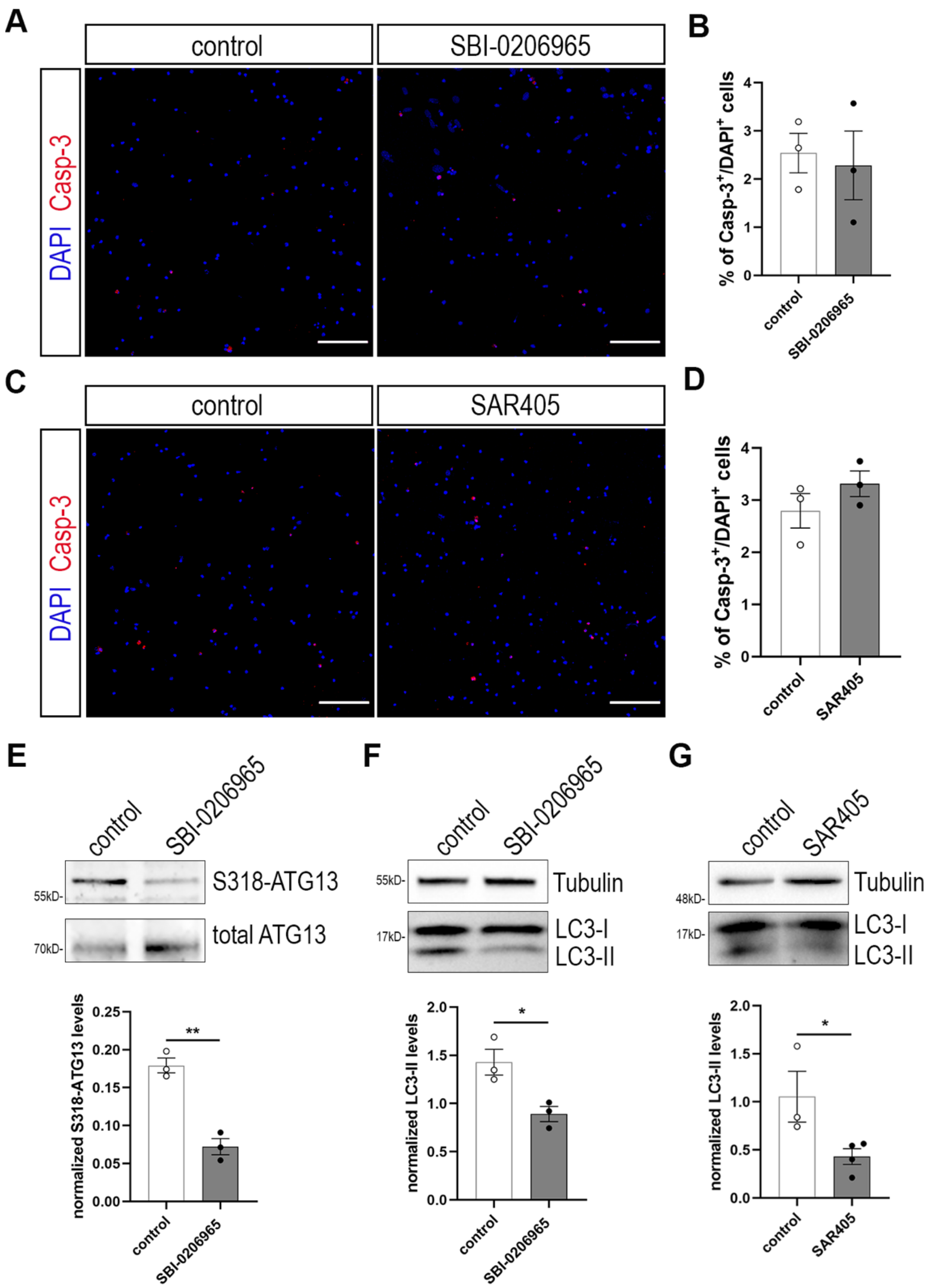

FIGURE S1: Inhibitors of autophagy do not cause apoptotic death in OLs. (A) Representative confocal images of DIV5 primary OLs, immunolabeled for cleaved caspase-3 (Casp-3, in red) and DAPI (in blue). Cells were either vehicle treated (control), or treated for 5 days with 1μM SBI-0206965. (B) Quantification of the percentage of the Casp-3-positive nuclei expressed as percentage of the total number of nuclei. (C) Representative confocal images of DIV5 primary OLs, immunolabeled for cleaved caspase-3 (Casp-3, in red) and DAPI (in blue). Cells were either vehicle treated (control), or treated for 5 days with SAR405 (1μM). (D) Quantification of the percentage of the Casp-3-positive nuclei expressed as percentage of the total number of nuclei. (E) Western blot analysis (top) and quantification (bottom) of DIV5 primary OLs with an antibody against S318-ATG13, and normalized for total-ATG13. Cells were either vehicle treated (control) or treated with SBI-0206965 (1μM) for 5 days. The medium was renewed 2 h prior to lysis. (F) Western blot analysis (top) and quantification (bottom) of DIV5 primary OLs with an antibody against LC3. Cells were either vehicle treated (control) or treated with SBI-0206965 (1μM) for 5 days. The medium was renewed 2 h prior to lysis. (G) Western blot analysis (top) and quantification (bottom) of DIV5 primary OLs with an antibody against LC3. Cells were either vehicle treated (control) or treated with SAR405 (1μM) for 5 days. The medium was renewed 2 h prior to lysis. Data information: Bars represent mean ± SEM. N=3 independent experiments per treatment. Student's t-test was used to determine statistical significance. Scale bars: 100 μm. $*p < 0.05$. $**p \leq 0.01$.

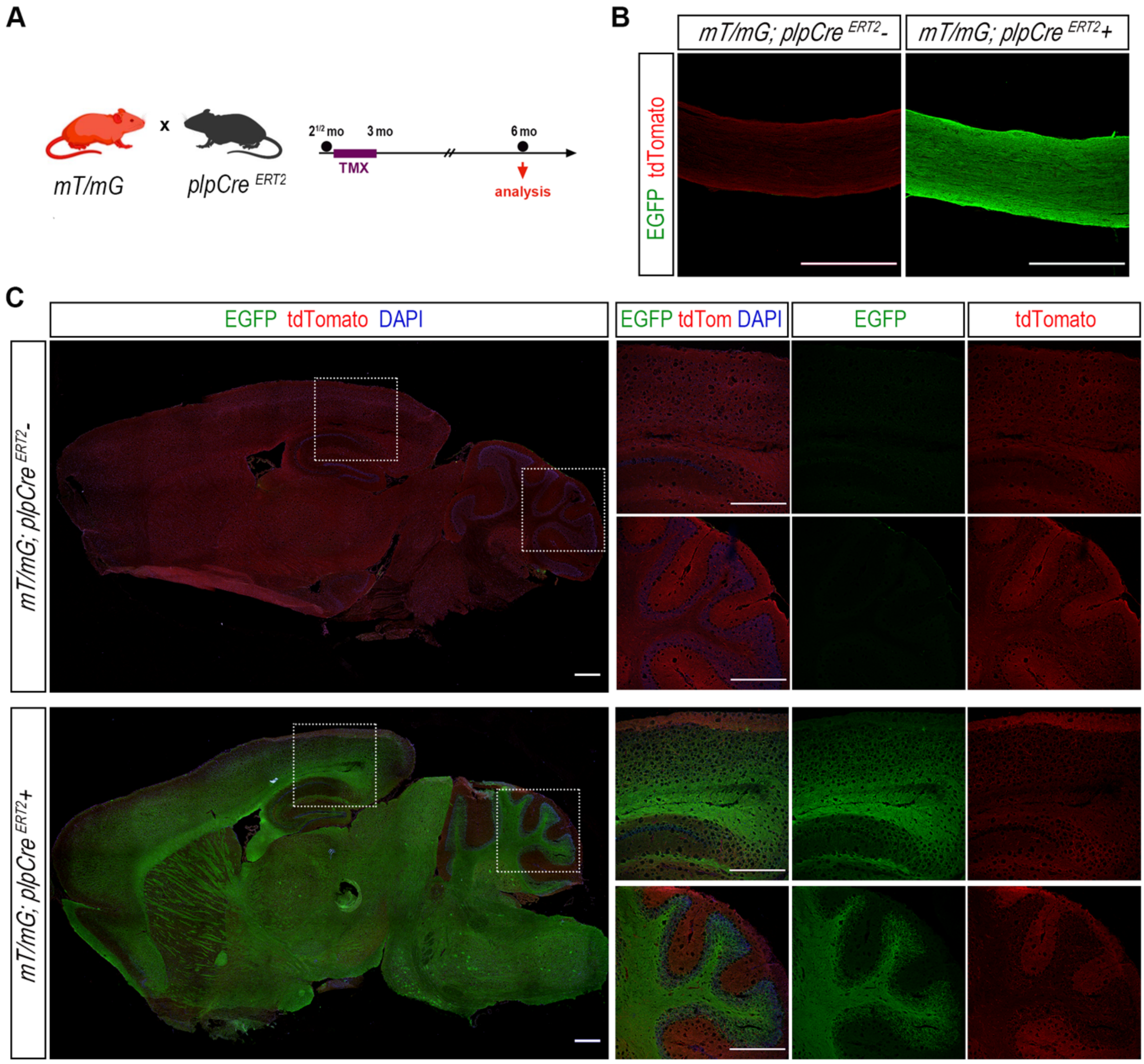

FIGURE S2: Recombination efficiency of tamoxifen inducible *plpCre*^{ERT2} line. (A) Schematic illustration of the experimental protocol used for TMX induction and analysis in the *plpCre*^{ERT2}*; atg5* $^{f/f}$ mice. All animals were injected i.p. with 1 mg of TMX per day at the age of 2.5 months for 10 days with two days break in between. The analysis was performed at 6 months of age. (B) Representative images of the percentage of GFP+ (green) recombinant cells in optic nerve cryosections of *mT/mG; plpCre*^{ERT2}+ and *mT/mG; plpCre*^{ERT2}- mice. Intense green fluorescent is detected only in *plpCre+* optic nerves at both ages. (C) Recombination efficiency in sagittal brain cryosections of 6mo *mT/mG; plpCre*^{ERT2}+ and *mT/mG*$^{+/-}$*; plpCre*^{ERT2}- mice which have received 10 doses of 1mg of TMX at 2.5 mo. Confocal images of the percentage of EGFP+ (green) recombinant cells (nuclei are stained with DAPI in blue) reveals green fluorescent in myelin tracts only in *plpCre+* transgenic mice. Rectangular boxes indicate two different representative areas (corpus callosum, cerebellum) magnified to the right. Data information: Scale bars: 500 µm.

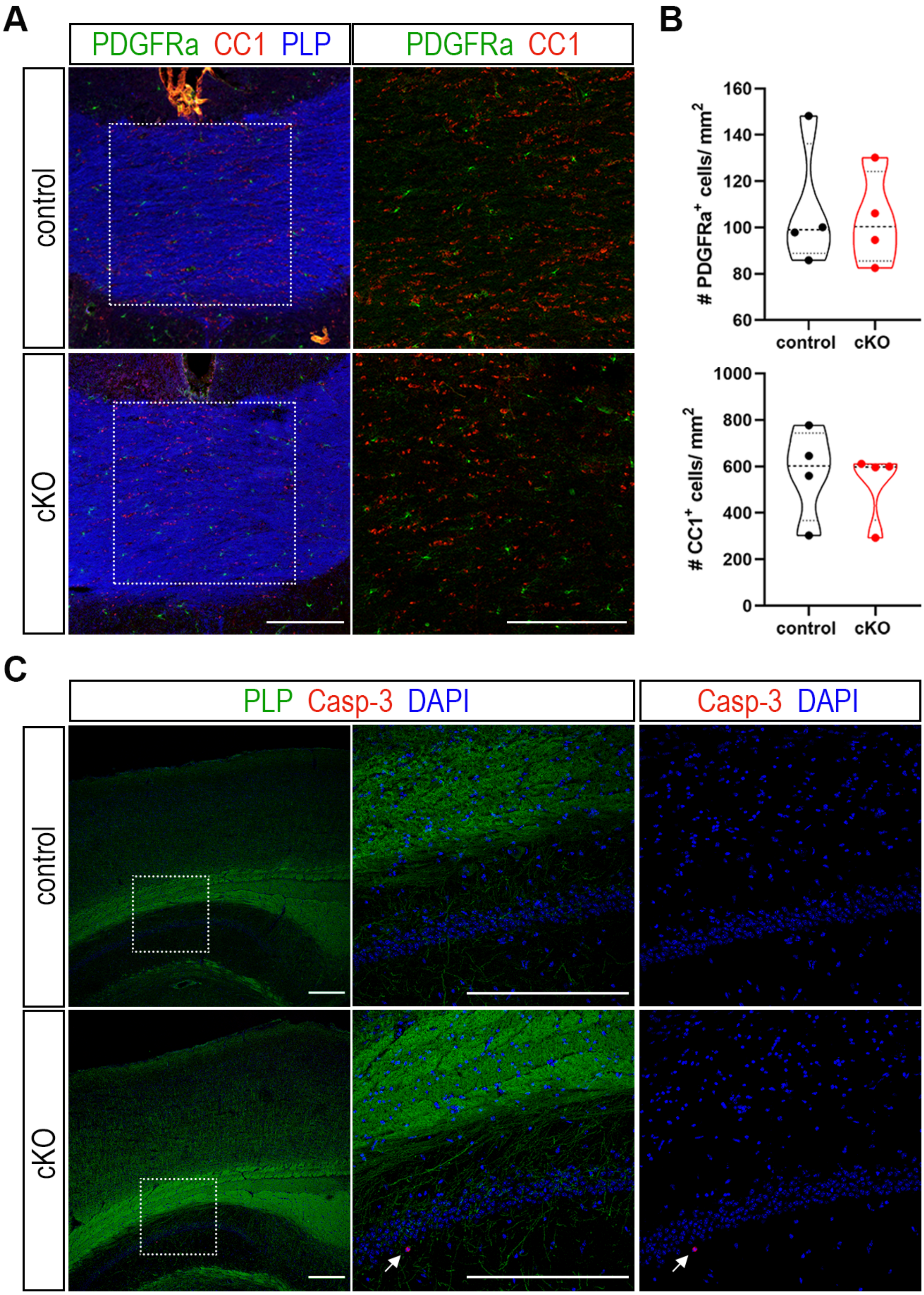

FIGURE S3: ATG5 is dispensable for OL survival. (A) Immunohistochemical analysis of rostral corpus callosum (cc) cryosections from 6 mo transgenic and control mice. The area of cc is recognized with immunolabeling for PLP (in blue). Two OL lineage markers were used: PDGFRa (in green) for OPCs and CC1 (in red) for mature OLs. (B) Quantification of PDGFRa+ OPCs and CC1+ mature OLs in cc. (C) Representative confocal images of sagittal cc sections from control and cKO mice immunolabeled for PLP (in green), cleaved Caspase-3 (Casp-3, in red) and DAPI (in blue). Rectangular boxes indicate areas magnified to the right. White arrows indicate a Casp3-positive cell. Data information: N=4 animals per genotype; Student's t-test was used to determine statistical significance. Scale bars: 200μm.

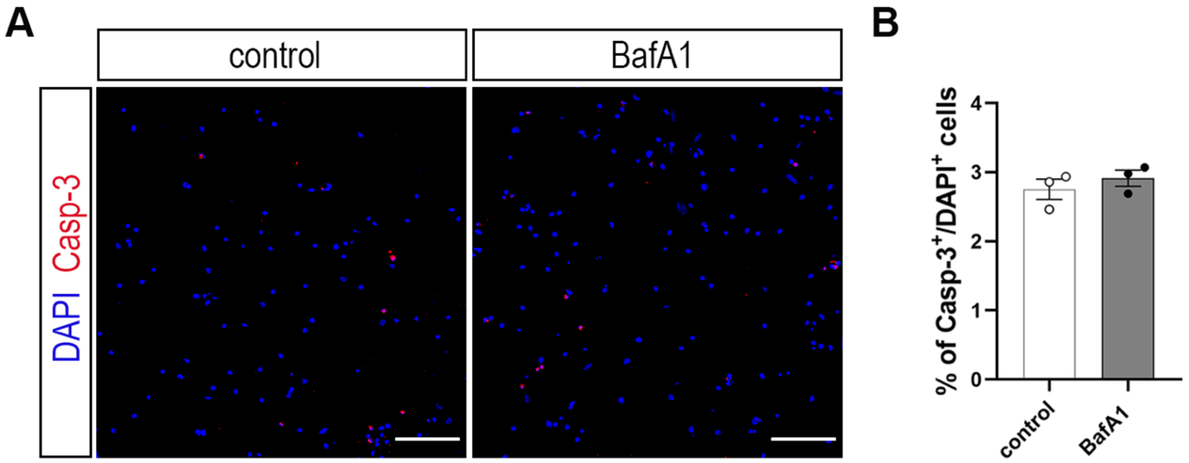

FIGURE S4: BafilomycinA1 treatment does not lead to increased apoptotic death. (A) Representative confocal images of DIV2 primary OLs, immunolabeled for cleaved caspase-3 (Casp-3, in red) and DAPI (in blue). Cells were either vehicle treated (control), or treated for 4 hr with 10nM BafilomycinA1 (BafA1). Scale bars: 100 µm. (B) Quantification of the percentage of the Casp-3-positive nuclei expressed as percentage of the total number of nuclei. Data information: Data are shown as mean ± SEM. Three independent experiments were used. Student's t-test was used to determine statistical significance. Scale bars: 100 µm.

TABLE S1: LIR motif prediction for PLP and MBP proteins. LIR motif prediction in PLP and MBP myelin proteins using iLIR software (https://ilir.warwick.ac.uk/). Table presents: the Uniprot ID of each isoform, the identified (WxxL) LIR motifs, the number of motifs in each isoform, the number of the amino acid residing on the start and end of each motif and the amino acid sequence of each motif. In addition, each motif is associated with a position-specific scoring matrix (PSSM) based on experimentally validated LIR motifs and the existence of intrinsically disordered protein regions as predicted by the ANCHOR package. Only the isoforms of MBP and PLP proteins with LIR motif prediction in *Mus musculus* are included in the table.

Protein name	Uniprot ID	Motif	# Motif	Start	End	Pattern	PSSM score	LIR in Anchor
PLP	P60202-1	WxxL	9	14	19	APFASL	4	No
				24	29	LCFFGV	-1	No
				56	61	QDYEYL	14	No
				66	71	HAFQYV	3	No
				76	81	ASFFFL	8	No
				150	155	DKFVGI	10	No
				161	166	VVWLLV	10	No
				205	210	RMYGVL	9	No
				263	268	YNFAVL	5	No
DM-20	P60202-2	WxxL	9	14	19	APFASL	4	No
				24	29	LCFFGV	-1	No
				56	61	QDYEYL	14	No
				66	71	HAFQYV	3	No
				76	81	ASFFFL	8	No
				115	120	ATFVGI	9	No
				126	131	VVWLLV	10	No
				170	175	RMYGVL	9	No
				228	233	YNFAVL	5	No
MBP	P04370-1	WxxL	2	197	202	THYGSL	3	Yes
				219	224	HFFKNI	-1	Yes
MBP- Isoform 4	P04370-4	WxxL	2	90	95	THYGSL	3	No
				112	117	HFFKNI	-1	Yes
MBP- Isoform 5	P04370-5	WxxL	2	64	69	THYGSL	3	Yes
				86	91	HFFKNI	-1	Yes
MBP- Isoform 6	P04370-6	WxxL	2	90	95	THYGSL	3	Yes
				112	117	HFFKNI	-1	Yes
MBP- Isoform 7	P04370-7	WxxL	2	64	69	THYGSL	3	Yes
				86	91	HFFKNI	-1	Yes
MBP- Isoform 8	P04370-8	WxxL	2	64	69	THYGSL	3	Yes
				86	91	HFFKNI	-1	Yes
MBP- Isoform 9	P04370-9	WxxL	2	90	95	THYGSL	3	Yes
				112	117	HFFKNI	-1	Yes
MBP- Isoform 10	P04370-10	WxxL	2	64	69	THYGSL	3	Yes
				86	91	HFFKNI	-1	Yes
MBP- Isoform 11	P04370-11	WxxL	2	90	95	THYGSL	3	Yes
				112	117	HFFKNI	-1	Yes
MBP- Isoform 12	P04370-13	WxxL	2	90	95	THYGSL	3	Yes
				112	117	HFFKNI	-1	Yes
MBP- Isoform 13	P04370-14	WxxL	2	64	69	THYGSL	3	Yes
				86	91	HFFKNI	-1	Yes
